# Symptom-Oriented, Connectome-Informed Deep Brain Stimulation for Asymmetric Dystonic Tremor: Unilateral Ventral Intermediate Nucleus (VIM) DBS Targeting a Tremor-Dominant Network

**DOI:** 10.3390/jcm15041666

**Published:** 2026-02-23

**Authors:** Olga Mateo-Sierra, Javier Ricardo Pérez-Sánchez, Beatriz De la Casa-Fages, María Teresa Del Castillo, Pilar Fernández, Pascual Elvira, José Paz, Francisco Grandas

**Affiliations:** 1 Department of Neurosurgery, Gregorio Marañón University Hospital, C/Dr Esquerdo 46, 28007 Madrid, Spain; 2 Gregorio Marañón Research Institute, Gregorio Marañón University General Hospital, C/Dr Esquerdo 46, 28007 Madrid, Spain; 3 Department of Surgery, Medicine School, Complutense University of Madrid, 28040 Madrid, Spain; 4 Movement Disorders Unit, Department of Neurology, Gregorio Marañón University Hospital, C/Dr Esquerdo 46, 28007 Madrid, Spain; 5 Department of Anesthesiology, Gregorio Marañón University Hospital, C/Dr Esquerdo 46, 28007 Madrid, Spain; 6 Department of Radiology, Gregorio Marañón University Hospital, C/Dr Esquerdo 46, 28007 Madrid, Spain; 7 Department of Anesthesiology, Dr Balmis University General Hospital, Av. Pintor Baeza 12, 03010 Alicante, Spain; 8 Department of Medicine, Medicine School, Complutense University of Madrid, 28040 Madrid, Spain

**Keywords:** deep brain stimulation, dystonic tremor, ventral intermediate nucleus (VIM), cerebellothalamic circuit, symptom-oriented DBS, connectome-informed targeting, neurorehabilitation

## Abstract

**Background:** Deep brain stimulation (DBS) has traditionally followed diagnosis-driven, nucleus-centered targeting paradigms. Increasing evidence supports a circuit-based framework in which clinical outcomes depend on modulation of symptom-relevant networks rather than diagnostic labels alone. This approach is particularly relevant in mixed movement disorder phenotypes such as dystonic tremor, where the most disabling symptom may not align with the conventional surgical target. **Methods:** We report a clinically illustrative single case treated using a symptom-oriented, connectome-informed DBS strategy. Clinical phenotype, tremor severity, functional impairment, prior medical and botulinum toxin treatments, and longitudinal outcomes were systematically reviewed. DBS target selection prioritized the dominant, treatment-refractory symptom rather than the underlying dystonia diagnosis. Surgical planning incorporated high-resolution MRI with patient-specific thalamic segmentation using Brainlab Brain Elements^®^, followed by postoperative lead localization and volume of tissue activated visualization with the SureTune™ platform. **Results:** A 54-year-old left-handed woman with long-standing cervical dystonia developed a severe, markedly asymmetric dystonic tremor predominantly affecting the left upper limb, resulting in profound functional disability. Instead of conventional bilateral globus pallidus internus DBS, unilateral right ventral intermediate nucleus (VIM) DBS was selected to engage tremor-related cerebellothalamic circuits. Rapid and marked improvement was observed, with tremor severity reduced to mild levels within 15 days after stimulation onset. At 6-month follow-up, overall tremor severity improved from 49 to 13 points on the Fahn–Tolosa–Marin Tremor Rating Scale, corresponding to a 73.5% reduction. This improvement was associated with restoration of legible handwriting, independent feeding and drinking, and recovery of bimanual fine motor function. Clinical benefit remained stable throughout follow-up, without stimulation-related adverse effects. **Conclusions:** This case illustrates the feasibility of a symptom-oriented, connectome-informed DBS strategy in selected patients with dystonic tremor. When symptom expression and network involvement are markedly asymmetric, selective unilateral modulation of the tremor-dominant circuit may achieve meaningful and durable functional improvement. Further studies are needed to assess the generalizability of this approach.

## 1. Introduction

Deep brain stimulation (DBS) is an established therapeutic option for medication-refractory movement disorders and provides sustained symptomatic improvement in selected patients with tremor, Parkinson’s disease, and dystonia [1,2]. Historically, DBS has followed nucleus-centered paradigms that link specific anatomical targets to diagnostic categories. Within this framework, globus pallidus internus (GPi) stimulation has been considered the standard target for dystonia, whereas stimulation of the ventral thalamic nucleus—particularly the ventral thalamic intermedius nucleus (VIM)—has been primarily reserved for tremor syndromes [3,4].

Over the past decades, advances in structural and functional connectomics have progressively reframed DBS as a network-level intervention. In this context, clinical outcomes depend less on modulation of a single anatomical nucleus and more on engagement of symptom-relevant neural circuits [5,6,7]. From this perspective effective neuromodulation involves distributed basal ganglia–thalamo–cortical and cerebello–thalamo–cortical circuits. Pathway-specific contributions shape both therapeutic efficacy and adverse effects [7,8]. Accordingly, DBS targeting has evolved from anatomical proximity toward alignment with the dominant pathological circuit underlying the patient’s clinical phenotype, rather than the diagnostic category alone [6,9].

Early critical appraisals of DBS targeting already questioned rigid nucleus-based paradigms and emphasized the limited understanding of how stimulation modulates pathological circuits, anticipating current network-oriented frameworks [10]. This paradigm shift is particularly relevant in mixed movement disorder phenotypes such as dystonic tremor, in which dystonia and tremor coexist but may arise from partially overlapping yet distinct network dysfunctions [11,12,13]. Recent connectivity-based approaches, including machine learning analyses of functional networks, further support the involvement of shared basal ganglia and cerebello–thalamo–cortical circuits in essential and dystonic tremor. Notably, the relative weighting of these circuits differs across phenotypes [14].

Although VIM DBS has been shown to effectively suppress tremor components in dystonic syndromes, most reported approaches remain grounded in nucleus-based logic and frequently rely on bilateral stimulation or combined pallidal and thalamic targeting to achieve satisfactory tremor control [15,16]. Clinical asymmetry and interindividual variability in network organization further complicate DBS target selection and have emerged as key determinants of outcome, particularly in tremor syndromes [17]. In such cases, selective modulation of the dominant dysfunctional circuit may achieve substantial symptomatic improvement while reducing surgical invasiveness, hardware burden, and stimulation-related adverse effects [6,8].

In parallel, technological advances in imaging and surgical planning have expanded the feasibility of connectivity-informed targeting. High-field magnetic resonance imaging (MRI), diffusion-based tractography, and connectivity-driven thalamic segmentation now allow more accurate localization of tremor-related circuits. This is particularly relevant in patients with anatomical distortion or cerebral atrophy, where indirect atlas-based targeting may be unreliable [17,18]. When combined with intraoperative verification, postoperative lead reconstruction, volume of tissue activated (VTA) modeling, and directional stimulation technologies, these tools support a precision neuromodulation approach tailored to individual network architecture [19,20].

Here, we present a clinically illustrative case of asymmetric dystonic tremor treated using a symptom-oriented and connectome-informed DBS strategy. Prioritization of the tremor-dominant network over the underlying dystonia diagnosis led to selection of unilateral VIM DBS and rapid tremor suppression with restoration of motor function.

## 2. Clinical Case and Targeting Strategy

A 54-year-old left-handed woman with a long-standing history of cervical dystonia was referred for evaluation because of progressive, disabling tremor refractory to optimized conservative management. The dystonic phenotype predominantly affected the cervical region, with tonic posturing and intermittent spasms that had remained reasonably controlled over time with regular botulinum toxin injections. In contrast, the patient developed a severe and markedly asymmetric dystonic tremor involving the left upper limb and a milder head–neck component, which progressively became the primary source of clinical disability.

Preoperatively, tremor showed a mixed postural and action component and significantly interfered with activities of daily living requiring fine motor control, including writing, bimanual coordination, and precision tasks essential to both professional and personal activities. Baseline tremor severity was severe on standardized clinical grading (rest 2/4; postural 3/4; action 3/4), with illegible handwriting and markedly abnormal spiral drawing (3/4). Functional impairment was quantified using the Fahn–Tolosa–Marin Tremor Rating Scale (FTM), with a baseline total score of 49 (severity 17, motor tasks 18, functional disability 14).

Despite optimized pharmacological therapy, targeted botulinum toxin injections, and participation in structured rehabilitation programs, the tremor remained poorly controlled, whereas tonic dystonic features were clinically stable and acceptable. Preoperative medication included zonisamide 50 mg twice daily and clonazepam 0.5 mg three times daily. Botulinum toxin therapy was continued at 300 IU every three months (left sternocleidomastoid 60 IU, left trapezius 70 IU, left splenius capitis 70 IU, left levator scapulae 30 IU, right masseter 20 IU, left masseter 20 IU, and right extensor hallucis longus 30 IU).

The case was discussed within a multidisciplinary movement disorders team comprising neurology, functional neurosurgery, neuroradiology, and rehabilitation specialists. Although GPi DBS would represent the conventional surgical target based on the underlying dystonia diagnosis, the tremor was identified as the dominant, treatment-refractory symptom driving disability. On this basis, DBS target selection was guided by symptom hierarchy and presumed circuit involvement rather than by diagnostic categorization alone.

Given the predominance of tremor and its marked asymmetry, a thalamic target was favored to modulate cerebellothalamic circuits implicated in tremor generation. VIM was selected as the primary target to engage tremor-related cerebellothalamic pathways, consistent with a network-oriented targeting strategy. The decision to pursue unilateral DBS was further supported by the lateralized clinical presentation, with the most disabling symptoms affecting the left upper limb and dominant hand, suggesting dominant right-hemispheric network involvement.

In this context, the surgical strategy was conceived as part of an integrated, multimodal therapeutic framework rather than as an isolated intervention. Residual tonic dystonic features were expected to remain amenable to ongoing botulinum toxin treatment, while DBS was intended to selectively suppress the tremor-dominant network that had proven refractory to non-surgical therapies. This approach aimed to maximize improvement in daily functioning while avoiding unnecessary bilateral implantation or combined pallidal and thalamic targeting at the initial stage.

**Figure 1:**
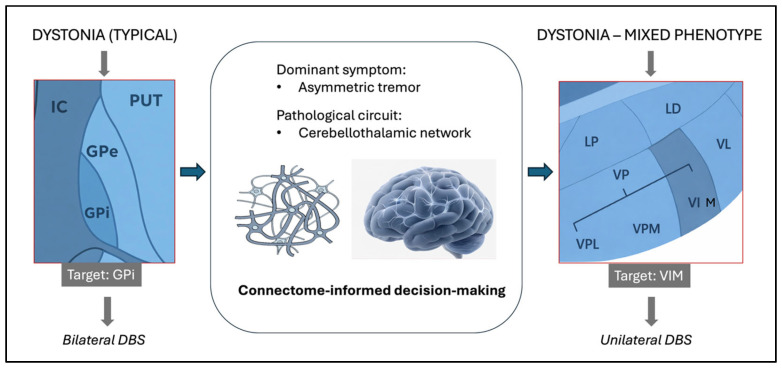
Conceptual illustration comparing a diagnosis-driven DBS targeting strategy with a symptom-oriented, circuit-based approach. While diagnosis-driven paradigms typically associate dystonia with bilateral globus pallidus internus (GPi) stimulation, a symptom-oriented strategy prioritizes modulation of the dominant dysfunctional network. In the present case, tremor-dominant cerebellothalamic circuitry was selectively targeted using unilateral ventral intermediate nucleus (VIM) DBS, reflecting symptom hierarchy and network involvement rather than diagnostic category alone.

In line with this rationale, the decision to perform unilateral right-sided VIM DBS reflected a symptom-oriented, circuit-based strategy tailored to the patient’s asymmetric clinical phenotype, dominant dysfunctional circuit, and overall therapeutic goals, with an emphasis on motor recovery and minimization of surgical burden (Figure 1).

## 3. Surgical Planning, Stimulation, and Outcome

Surgical planning was performed using high-field 3 Tesla MRI within a multimodal stereotactic workflow that routinely incorporates advanced image fusion and patient-specific thalamic segmentation using Brainlab Brain Elements^®^ (Brainlab, Munich, Germany). Preoperative imaging included contrast-enhanced 3D T1-weighted inversion recovery sequences of the basal ganglia, as well as 3D T2-weighted and 3D T2-FLAIR sequences, all of which were coregistered within the planning environment.

**Figure 2:**
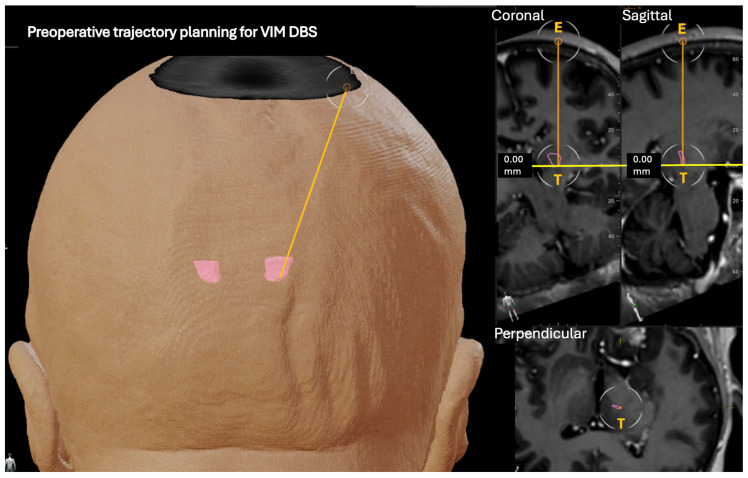
Preoperative DBS planning using the Brainlab Brain Elements^®^ platform. Patient-specific thalamic segmentation and trajectory planning are shown. The final VIM target (pink) and surgical trajectory were refined based on individual anatomy and segmentation rather than indirect atlas-based coordinates, allowing alignment with tremor-related cerebellothalamic pathways.

Given the known interindividual variability of thalamic anatomy, a combined targeting strategy integrating conventional indirect atlas-based coordinates with patient-specific anatomical segmentation was employed, in line with our standard surgical practice. In this patient, anatomical variability—further accentuated by the presence of cerebral atrophy—highlighted the limitations of relying on indirect targeting alone. While indirect VIM coordinates were initially estimated as a reference, segmentation-based visualization allowed refinement of both target location and surgical trajectory. This integrated approach identified a functionally relevant VIM position that differed from classical indirect atlas-based coordinates and was more closely aligned with tremor-related cerebellothalamic pathways (Figure 2).

Surgery was performed under awake conditions with intermittent sedation using a frame-based stereotactic approach with a Leksell Vantage stereotactic frame. Intraoperative neurophysiological monitoring supported target verification and optimization of lead placement and was complemented by micro- and macrostimulation testing to functionally confirm the target location. Intraoperative stimulation elicited a robust antitremor response, with capsular side effects detected only at higher stimulation intensities, supporting appropriate target localization with a favorable therapeutic window.

Trajectory planning was individualized to account for anatomical variability, with careful avoidance of eloquent structures and vascular elements while maximizing engagement of the intended tremor-related network. Directional SenSight™ DBS leads (Medtronic, Minneapolis, MN, USA) were implanted at the planned target location to preserve flexibility for postoperative current steering.

**Figure 3:**
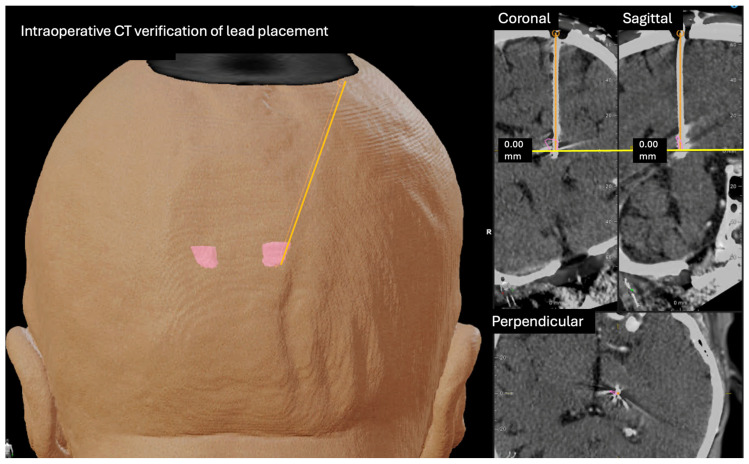
Postoperative verification of DBS lead placement. Fusion of preoperative magnetic resonance imaging with intraoperative computed tomography acquired using the Loop-X system demonstrates accurate electrode positioning along the planned trajectory and concordance with the intended VIM target. Axial, coronal, and sagittal views are shown.

Intraoperative computed tomography was subsequently performed to verify accurate lead placement. CT acquisition and fusion with the preoperative magnetic resonance imaging were carried out using the Loop-X system (Brainlab AG, Munich, Germany), enabling three-dimensional reconstruction of electrode location relative to the intended target (Figure 3) and confirming concordance between planned and implanted trajectories.

Following radiological confirmation of lead position, a rechargeable implantable pulse generator was implanted according to standard institutional protocols. No intraoperative or early postoperative complications occurred. Postoperative recovery was uneventful, and the patient was discharged in stable condition.

**Figure 4:**
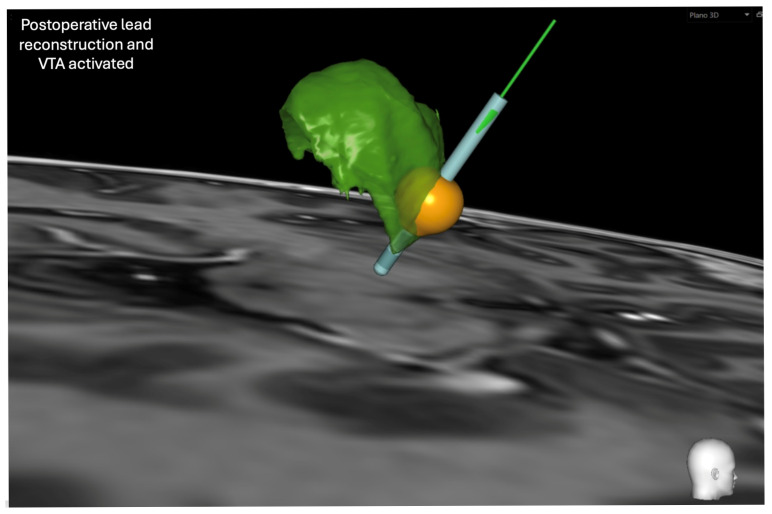
Postoperative lead reconstruction and volume of tissue activated (VTA) modeling using the SureTune™ platform. The active contact and corresponding VTA are shown in relation to the segmented ventral intermediate nucleus (VIM), displayed in green. The VTA is predominantly confined to the lateral VIM, consistent with effective engagement of tremor-related cerebellothalamic pathways. Clinically effective tremor control was achieved at moderate stimulation amplitudes, with capsular side effects observed only at higher intensities.

Stimulation was initiated 15 days after surgery. Postoperative programming was guided by lead reconstruction and volume of tissue activated (VTA) modeling using the SureTune™ platform (Medtronic, Minneapolis, MN, USA) to align stimulation parameters with the targeted cerebellothalamic network (Figure 4). Chronic stimulation was delivered using contact 11 (−) with the case as anode (C+), pulse width 60 μs, and frequency 125 Hz. Amplitude was initiated at 2.5 mA and gradually increased to 3.3 mA over the first year, after which it remained stable. Following stimulation onset, tremor severity decreased substantially. At 15 days, tremor was graded as 1/4 across rest, postural, and action components, and handwriting became legible. At 6-month follow-up, tremor severity improved from 49 to 13 points on the Fahn–Tolosa–Marin Tremor Rating Scale (FTM) (severity 5, motor tasks 6, functional disability 2), corresponding to a 73.5% reduction. Clinical improvement translated into restoration of fine motor control and bimanual task performance, including independent feeding and drinking. Follow-up visits were performed at 15 days, 1 month, 2 months, 4 months, 8 months, and 12 months, with sustained benefit throughout.

Owing to the accurate electrode location and the favorable therapeutic response achieved with conventional stimulation settings, use of directional current steering was not required, although directional leads had been implanted to preserve programming flexibility if needed. This finding further supports the relevance of precise anatomical targeting in achieving robust clinical benefit.

No stimulation-related adverse effects were observed during follow-up. Residual tonic dystonic features of the cervical region remained stable and continued to respond satisfactorily to adjunctive botulinum toxin therapy, without the need for additional surgical intervention. Following DBS, tremor improvement allowed reduction in zonisamide dosage to 50 mg once daily and clonazepam to 0.5 mg once daily, while botulinum toxin dosing and distribution remained unchanged.

## 4. Discussion

This case illustrates a symptom-oriented, connectome-informed approach to DBS target selection in a complex movement disorder phenotype. It highlights how prioritization of the dominant dysfunctional circuit, rather than diagnostic categorization alone, can yield robust and clinically meaningful outcomes [6,9]. Although GPi stimulation remains the conventional target for dystonia, this case demonstrates that VIM DBS may represent an effective and sufficient strategy when tremor constitutes the primary driver of disability, even in patients with an underlying dystonic disorder [3,15].

### 4.1. From Diagnosis-Driven to Symptom-Driven DBS Targeting

Traditional DBS paradigms have largely relied on diagnosis-based target assignment. While effective at the population level, this approach may lack flexibility in mixed or atypical phenotypes [2,21]. Dystonic tremor exemplifies this limitation, as it reflects the convergence of pallidothalamic and cerebellothalamic network dysfunctions with variable weighting across individuals [12]. In such scenarios, a diagnosis-driven strategy may fail to adequately address the symptom that most directly determines functional impairment.

In the present patient, tonic cervical dystonia was reasonably controlled with botulinum toxin therapy, whereas the tremor component affecting the left upper limb and head–neck region was severe, markedly asymmetric, and refractory to optimized conservative management. This clear symptom hierarchy supported prioritization of tremor-related circuitry over pallidal targets. The favorable outcome reinforces a decision-making model in which DBS target selection is guided by the dominant symptom and its presumed circuit substrate rather than by diagnostic category alone [6].

### 4.2. Circuit-Based Rationale for VIM DBS in Dystonic Tremor

Accumulating connectomic and electrophysiological evidence indicates that dystonic tremor involves both basal ganglia–thalamo–cortical and cerebellothalamic networks, with the latter playing a central role in tremor generation [12,18]. Thalamic targets, particularly VIM and adjacent regions, have therefore been explored for tremor suppression in dystonic syndromes, with several series reporting clinically relevant improvement [15,22].

Recent functional connectivity studies using advanced network-based and graph-theoretical models further support the concept that dystonic tremor arises from dysfunction within the canonical tremor circuitry, with variable contributions of cerebellothalamic and basal ganglia networks across patients [14]. In contrast to conventional nucleus-centered approaches, the present case was explicitly framed within a circuit-based paradigm, in which connectome-informed planning identified a functionally relevant VIM position aligned with tremor-related cerebellothalamic pathways rather than with indirect atlas-based coordinates.

Engagement of symptom-relevant pathways, rather than proximity to anatomical nuclei alone, has been shown to be a key determinant of DBS efficacy [5,7]. This refined functional targeting likely contributed to rapid, consistent, and durable tremor control observed in this patient.

### 4.3. Unilateral Versus Bilateral DBS: Symptom Hierarchy and Surgical Efficiency

The choice between unilateral and bilateral deep brain stimulation (DBS) remains a central consideration in the surgical management of tremor and dystonia [18]. Bilateral stimulation is frequently advocated to address axial or head tremor and to maximize symptom control in complex phenotypes; however, it also increases surgical invasiveness, hardware burden, programming complexity, and the risk of stimulation-related adverse effects [23,24,25].

Several series have reported favorable outcomes with bilateral or combined targeting strategies in dystonic tremor, including thalamic and pallidal approaches, albeit often at the cost of greater procedural complexity and long-term management demands [1,2,3]. Accordingly, reported DBS strategies for dystonic tremor vary substantially in terms of target selection and laterality, as summarized in Table 1. Comparative data further suggest that clinical benefit depends less on diagnostic category than on effective engagement of symptom-relevant networks [4,5].

Clinical asymmetry played a central role in shaping the surgical strategy in the present case. The marked lateralization of tremor affecting the left upper limb and head–neck region suggested dominant right-hemispheric network involvement, supporting selective unilateral modulation of the tremor-dominant cerebellothalamic circuit [18]. In this context, unilateral DBS was sufficient to achieve rapid and sustained clinical improvement without the need for bilateral stimulation or combined targeting, consistent with observations from recent comparative studies [6].

A central aspect of the surgical strategy was that unilateral DBS should not be viewed as a simplified or inferior option, but rather as a tailored approach guided by symptom hierarchy and network involvement. From a clinical perspective, this strategy may reduce surgical invasiveness, hardware burden, and the risk of stimulation-related adverse effects in carefully selected patients. In this context, a staged approach—beginning with unilateral stimulation and reserving bilateral implantation for symptom progression or bilateral network involvement—may represent a pragmatic and patient-centered strategy.

### 4.4. Precision Targeting and Programming Efficiency in Anatomically Variable Brains

Advanced imaging and connectome-informed planning played a central role in this patient, in whom cerebral atrophy limited the reliability of indirect atlas-based targeting. Although indirect stereotactic coordinates remain a useful reference, multiple studies have demonstrated substantial interindividual variability in the optimal mediolateral and anteroposterior location of the ventral intermediate nucleus (VIM), particularly when ventricular size and thalamic morphology deviate from standard assumptions [17,18,26]. Individualized thalamic segmentation therefore enabled a refined definition of target location and trajectory aligned with symptom-relevant circuits rather than with fixed anatomical coordinates [7,17,18].

In the present case, the final active contact was located slightly more medial than predicted by classical ventricular adjustment formulas. Nevertheless, postoperative reconstruction demonstrated that the volume of tissue activated intersected the lateral portion of the segmented VIM, a region considered anatomically congruent with the expected course of the dentatorubrothalamic tract (DRTT) according to atlas-based descriptions [17,18,26]. This finding supports growing evidence that effective tremor suppression does not necessarily require stimulation of the geometric center of the VIM, but rather engagement of functionally relevant subregions or adjacent pathways [7,19].

An additional implication of this case relates to programming efficiency and network-specific modulation. Effective tremor control was achieved using a single, well-localized contact without the need for complex directional current steering or extensive parameter adjustments over time. After a modest amplitude increase during early follow-up, stimulation settings remained stable throughout the first year, suggesting consistent engagement of the targeted cerebellothalamic network.

This stability contrasts with trial-and-error programming strategies often required when targeting is suboptimal or when stimulation engages competing networks. The rapid clinical response, minimal programming burden, and absence of stimulation-related adverse effects support the concept that precise, anatomically and functionally informed targeting can enhance therapeutic efficiency [7,8,19].

Overall, these findings suggest that the functionally optimal target for tremor suppression may differ from classical geometric VIM coordinates, particularly in patients with anatomical variability, and that advanced anatomical planning combined with functional validation can reliably identify an effective stimulation site.

### 4.5. Integrated Therapy and Functional Recovery

Finally, this case underscores the importance of integrating DBS into a broader multimodal therapeutic framework. Rather than addressing all symptom components surgically, DBS was used selectively to suppress the tremor-dominant network, while residual dystonic features remained effectively managed with botulinum toxin therapy.

Crucially, the observed benefit cannot be attributed to concurrent changes in medical or botulinum toxin therapy, as pharmacological treatment was reduced and botulinum toxin dosing and distribution remained stable during follow-up. The rapid recovery of fine motor control and the patient’s ability to resume high-precision bimanual activities suggest that stabilization of pathological network activity through DBS may provide a favorable substrate for rehabilitation-driven plasticity and functional restoration [27,28].

## 5. Limitations

This report is limited by its single-case design, which precludes generalization of the findings. Baseline tremor severity was documented using standardized clinical grading across tremor domains rather than a complete preoperative total score, limiting granular quantitative comparisons. In addition, no direct comparison with alternative surgical targets, such as globus pallidus internus DBS, was performed.

The symptom-oriented, unilateral approach described here may not be applicable to patients with symmetric tremor or more generalized dystonia. Nevertheless, the detailed clinical characterization, longitudinal follow-up, and integration of advanced imaging and connectome-informed planning provide valuable insights into circuit-based DBS strategies in carefully selected patients.

## 6. Conclusions

This case illustrates the feasibility of a symptom-oriented, connectome-informed strategy for deep brain stimulation in a patient with dystonic tremor. When symptom hierarchy and network involvement are clearly asymmetric, selective unilateral modulation of the tremor-dominant cerebellothalamic circuit can provide meaningful and durable functional improvement, even in the context of an underlying dystonic disorder.

Precise, patient-specific targeting, supported by advanced imaging and segmentation techniques, enabled effective tremor control with minimal programming complexity and without stimulation-related adverse effects. Of note, the observed clinical benefit was achieved independently of changes in concomitant medical or botulinum toxin therapy.

Although limited by its single-case design, this report supports the concept that DBS target selection guided by symptom dominance and circuit dysfunction may represent a valuable strategy in carefully selected patients. Further studies are required to assess the reproducibility and generalizability of this approach.

## Figures and Tables

**Table 1:** DBS Targets and Strategies Reported in Dystonic Tremor.

Study	Phenotype	DBS Target	Laterality	Main Outcome
Sobstyl et al., 2020 [24]	Cervical dystonia with severe tremor	GPi + VIM	Bilateral	Significant tremor reduction achieved with combined targeting, at the cost of increased surgical and programming complexity
Trompette et al., 2022 [16]	Dystonic tremor	GPi + VIM	Bilateral	Effective tremor control using combined pallidal and thalamic stimulation
Nagel et al., 2025 [15]	Dystonic head tremor	VIM	Mostly bilateral	Sustained tremor improvement with thalamic stimulation
Paschen et al., 2024 [25]	Dystonic and essential tremor	VIM vs. GPi	Unilateral or bilateral	Clinical outcome depends on symptom dominance and circuit involvement rather than diagnosis alone
Tsuboi et al., 2021 [12]	Dystonic vs. essential tremor	Network-based analysis	Unilateral or bilateral	Engagement of symptom-relevant networks predicts DBS outcome
Present case	Asymmetric dystonic tremor	VIM	Unilateral	73.5% reduction in FTM score with rapid and sustained functional improvement

## Data Availability

The data generated and analyzed during this study are not publicly available due to patient confidentiality but are available from the corresponding author upon reasonable request. Written informed consent for publication was obtained from the patient.

## Abbreviations

DBS	Deep brain stimulation
VIM	Ventral intermediate nucleus
GPi	Globus pallidus internus
FTM	Fahn–Tolosa–Marin tremor rating scale
MRI	Magnetic resonance imaging
VTA	Volume of tissue activated
